# Retinal safety and toxicity study of artesunate *in vitro* and *in vivo*

**DOI:** 10.1016/j.aopr.2022.11.003

**Published:** 2022-12-10

**Authors:** Bing-Wen Lu, Yu-Xiang Liang, Jin-Feng Liu, Zhong-Qing Sun, Kwok-Fai So, Kin Chiu

**Affiliations:** a Department of Ophthalmology, Li Ka Shing Faculty of Medicine, The University of Hong Kong, Hong Kong, China; b State Key Laboratory of Brain and Cognitive Sciences, The University of Hong Kong, Hong Kong, China; c Department of Psychology, Faculty of Social Sciences, The University of Hong Kong, Hong Kong, China; d Guangdong-Hongkong-Macau (GHM) Institute of CNS Regeneration, Ministry of Education, CNS Regeneration Collaborative Joint Laboratory, Jinan University, Guangzhou, China

**Keywords:** Artesunate, Safety, Retina, Microglia

## Abstract

**Background:**

Artesunate (ART), a member of the artemisinin family, possesses multi-properties, including anti-inflammation, anti-oxidation, and anti-tumor. ART was recently reported to show anti-neovascularization effect on the cornea, iris, and retina. Compared to the expensive anti-VEGF treatment, this versatile, economical treatment option is attractive in the ophthalmic field. The safety and toxicity profile of ART intravitreal application are in utmost need.

**Methods:**

In this study, immortalized microglial (IMG) cells were treated with ART to determine the safe concentrations without inducing overt inflammatory reactions. Reverse transcription-polymerase chain reaction analysis was used to detect the cytokine expressions in IMG cells in response to ART stimulation. Various doses of ART were intravitreally injected into the right eyes of C57BL/6 mice. Retinal function was tested by electroretinogram, and retinal ganglion cell (RGC) survival was evaluated by counting Brn3a stained cells in flat-mounted retinas at 7 days after ART injection.

**Results:**

ART below 5μM was safe for IMG cells *in vitro*. Both 2.5 and 5 ​μM ART treatment increased IL-10 gene expression in IMG cells while not changing IL-1β, IL-6, TNF-α, and Arg-1. In the *in vivo* study, intravitreal injection of ART below 100 ​μM did not cause deterioration in the retinal function and RGC survival of the mouse eyes, while 1 ​mM ART treatment significantly attenuated both the scotopic and photopic b-wave amplitudes and impaired RGC survival. In addition, treatment with ART of 25, 50, and 100 ​μM significantly decreased TNF-α gene expression while ART of 100 ​μM significantly increased IL-10 in the mouse retina.

**Conclusions:**

Intravitreal injection of 100 ​μM ART could downregulate TNF-α while upregulate IL-10 in the mouse retina without causing retinal functional deterioration and RGC loss. ART might be used as anti-inflammatory agent for retinal disorders.

## 1 Introduction

Artesunate (ART) is a semisynthetic, water-soluble, and widely used derivative of artemisinin. In addition to the antimalarial activities, ART was proved to possess remarkable potential in anti-oxidation, anti-inflammation, anti-tumor, and anti-angiogenesis.^1^^,^^2^ The versatile effects, the convenience for synthesis and storage, as well as the safety in systemic use enable ART to become a promising treatment candidate for various ocular disorders.

In ophthalmology, ART has been shown to have remarkable anti-angiogenic potentials demonstrated by neovascularization (NV) inhibition in rabbit models and alleviate macular edema (ME) in monkeys.^3^^,^^4^ A pilot clinical trial including 5 patients with retinal disorders has confirmed the efficacy of single intravitreal ART injection at 80 ​μg (about 46.2 ​μM) on the anti-NV effect.^5^ The safety evaluation and ART efficacy in their findings were exploratory because the subjects they included were of no light perception. A recent *in vitro* study using BV2 microglial cell line and N-2a neuronal cell line reported that ART at 1 ​μM significantly affected cell viability^6^ further challenged the dosage used *in vivo*. Conducting a study to evaluate the safety profile for its intravitreal use as an economical anti-NV agent would be necessary before further pre-clinical or clinical study.

The aim of this study is to provide evidence for the safety profiles of ART for its intra-ocular application. We conducted *in vitro* studies to find out a dose without inducing significant cell survival impairment and activation in microglial cells under ART challenge. Then we examined the safety of ART as an intravitreal agent through retinal functional analysis and retinal ganglion cell (RGC) survival evaluation.

## 2 Materials and methods

### 2.1 Cells and reagents

Immortalized microglial (IMG) cell line was purchased from Sigma-Aldrich (SCC134, St. Louis, MO, USA). The detailed characteristics of this cell line have been previously described by McCarthy et al.^7^ ART (Sigma-Aldrich; Merck KGaA, Darmstadt, Germany) was dissolved in a sodium bicarbonate solution and then diluted to different concentrations with the final pH at 7.2–7.4.

### 2.2 Cell culture and treatment

IMG cells were cultured in Dulbecco's modified Eagle's medium (DMEM) with high glucose (4.5 g/L) supplemented with fetal bovine serum (FBS) (10%) (Invitrogen, Carlsbad, CA, USA) and penicillin-streptomycin (1%), and incubated in the assigned atmosphere (95% O_2,_ 5% CO_2,_ 37 ​°C). The medium was renewed every two days. IMG cells at P5-7 were used for ART treatments. Cells were cultured with DMEM-low glucose medium and 1% (v/v) FBS. DMEM only were served as control group and the ART treatment groups were administrated with 1.25, 2.5, 5, 10, 20, and 50 ​μM ART and incubated for 24 ​h at 37 ​°C, 5% CO_2_. For lactate dehydrogenase (LDH) release assay and PrestoBlue assay, cells were seeded into 96-well plates (1 ​× ​10^4^ ​cells/well) and for Reverse transcription-polymerase chain reaction (RT-PCR) assessment, they were seeded in 12-well plates (15 ​× ​10^4^ ​cells/well).

### 2.3 Assessment of lactate dehydrogenase (LDH) release

LDH release was detected with a commercially available assay kit (88953, Thermo Fisher Scientific, Waltham, MA, USA) to evaluate cell toxicity. At 24 ​h after ART treatment, 100 ​μL cell culture medium was transferred to a 96-well plate. The reaction mixture (the LDH detection buffer) was then added to each well. After incubation for 30 ​min at room temperature, the stop solution was added into each well. The absorbance at 490 ​nm and 680 ​nm was detected with a spectrometry (EnSpire PerkinElmer, Waltham, MA, USA). The 490 ​nm absorbance value after subtraction of the 680 ​nm absorbance value (background signal) represented the LDH activity.

### 2.4 PrestoBlue assay

Cell viability was assessed with Prestoblue assay kit (A13261, Thermo Fisher Scientific, Waltham, MA, USA). After incubation in FBS-free medium with PrestoBlue for 2 h at 37 ​°C, the fluorescent activity at the wavelength of 570 nm was detected with a spectrometry (EnSpire PerkinElmer, Waltham, MA, USA).

### 2.5 Real-time polymerase chain reaction

Total RNAs of IMG cells were extracted with an RNA extraction kit (74106, QIAGEN, Hilden, Nordrhein-Westfalen, Germany). Afterward, they were reverse transcribed into cDNA with a commercially available kit (205413, QIAGEN, Hilden, Nordrhein-Westfalen, Germany). RT-PCR was performed with an SYBR Green PCR Kit (208056, QIAGEN, Hilden, Nordrhein-Westfalen, Germany). Primer sequences used were presented in Table 1. The reaction was performed with an ECO system (Gene Company Limited, HK, China) (30 s at 95 ​°C, 40 cycles of 5 s at 95 ​°C, and 15 s at 60 ​°C). The mRNA expression levels were normalized to that of β-actin.

**Table 1:** Primer sequences used for analyses.

Gene	Forward primer sequences	Reverse primer sequences
TNF-α	5-CCTGTAGCCCACGTCGTAG-3	5-GGGAGTAGACAAGGTACAACCC-3
IL-6	5-AGCCACTGCCTTCCCTAC-3	5-TTGCCATTGCACAACTCTT-3
IL-1β	5-CTGTGACTCATGGGATGATGATG-3	5-CGGAGCCTGTAGTGCAGTTG-3
IL-10	5-CTTACTGACTGGCATGAGGATCA-3	5-GCAGCTCTAGGAGCATGTGG-3
Arg-1	5-TTGGGTGGATGCTCACACTG-3	5-GTACACGATGTCTTTGGCAGA-3
β-actin	5-GTGACGTTGACATCCGTAAAGA-3	5-GCCGGACTCATCGTACTCC-3

Abbreviations: TNF, tumor necrosis factor; IL, interleukin; Arg, arginase.

### 2.6 Animals

A total of 30 C57BL/6 mice (male, 8-week-old) were obtained from the Laboratory Animal Unit, Li Ka Shing Faculty of Medicine, the University of Hong Kong. The mice were housed in concordance with bio-ethical requirements under specific pathogen-free condition, with 12:12 light:dark cycles, adequate ventilation, appropriate humidity (40–70%), and adequate access to food and clean water. All procedures followed the requirements of the ARVO Statement for the Use of Animals in Ophthalmic and Vision Research.

### 2.7 Intravitreal injection

Mice were anesthetized with a solution containing ketamine (120 ​mg/kg), xylazine (20 ​mg/kg), and saline solution (1:1:8) through intraperitoneal injection. Oxybuprocaine hydrochloride eye drops (Santen Pharmaceutical Co. Ltd., Osaka, Japan), tropicamide eye drops (Santen), and ofloxacin ophthalmic ointment (Shenyang Xingqi Pharmaceutical Co., Ltd., Shenyang, China) were applied for topical anesthesia, pupil dilation, and infection prevention, respectively.

For the retinal safety study, 30 mice were divided into 6 groups randomly, including one control group and five groups receiving ART of different concentrations. ART was diluted with sterile saline with final concentrations at 25, 50, 100 ​μM, and 1 ​mM. The micro-injector was inserted into the vitreous through the incision made by a 30-gauge needle 0.5 ​mm posterior to the limbus. Serially diluted ART solutions or sterile saline of 1 ​μL were then injected into the mouse eye under a surgical microscope. Ocular changes including intraocular inflammation, retinal hemorrhage and cataract were observed through daily ophthalmic examination after the injection.

### 2.8 Electroretinography

ERG recordings were performed as previously reported with a full-field flash ERG and an LED stimulator (Ganzfeld Color DomeTM, Diagnosys LLC, Littleton, MA).^8^ The mouse was placed on a temperature-controlled platform connected to the ERG system. ERG recordings were taken from both eyes using two custom-made gold ring electrodes (active electrodes) placed on the corneal surface, one ground electrode inserted into the base of the tail, and one reference electrode inserted into the subcutaneous of the scalp. All ERG records were conducted under dim red light. Scotopic ERGs were examined with three light flashes of increasing energy (0.01, 0.1, and 3.0 ​cd ​s/m^2^) after adequate dark adaption. Afterward, the mouse was exposed to flashes of light once every 5–10 ​s. Photopic ERGs were tested using flashes of 3.0 and 10.0 ​cd ​s/m^2^. ERG results were further analyzed using Espion E3 software (Diagnosys LLC, Lowell, MA). The a-wave amplitude was measured from the baseline to the a-wave trough, and the b-wave amplitude was measured from the a-wave trough to the b-wave peak.

### 2.9 Whole retinal flat mount and RGC count

The eyes were immediately enucleated after the ERG measurement and fixed with 4% paraformaldehyde (PFA) at 4 ​°C for 1 ​h before further dissection of the retina. Retina was dissected from sclera and post-fixed for another 4 ​h followed by rinsing with phosphate-buffered saline (PBS, 0.01 ​M, pH 7.4). Retinas were kept in PBS for 24 ​h before immunohistochemical study. Free floating retinas were incubated overnight at 4 ​°C with the primary antibody, anti-Brn3a (Santa Cruz Biotechnology), followed by secondary donkey anti-goat antibody (Alexa Fluor 488, Thermo Fisher Scientific) at room temperature for 2.5 ​h. Then the retina was flat mounted on the slide with RGC face up. 400x magnification images were captured every 500 ​μm from the center of the optic disc in four retinal quadrants using a EVOS M7000 Imaging System (Thermo Fisher Scientific) generating 16 images in total. ImageJ software was used to count the number of Brn-3a positive RGC cells in each image, and the average RGC density of all images was calculated and presented as mean RGCs density (RGCs/mm^2^) ​± ​standard deviation (SD) for each animal.

### 2.10 Statistical analysis

All data were expressed as the mean ​± ​SD. SPSS software (Version 17, SPSS, Inc., Chicago, IL; USA) was used for statistical analyses. One-way ANOVA was chosen for comparisons among groups and repeated measures for ERG data analyses. The experimental group and the control group were compared by *t*-test. Figures were generated using the Prism GraphPad software (Prism Inc. version 8.0). *P-*value ＜ 0.05 was regarded as statistically significant.

## 3 Results

### 3.1 Confirmation of the optimal concentration of ART *in vitro*

**Fig. 1:**
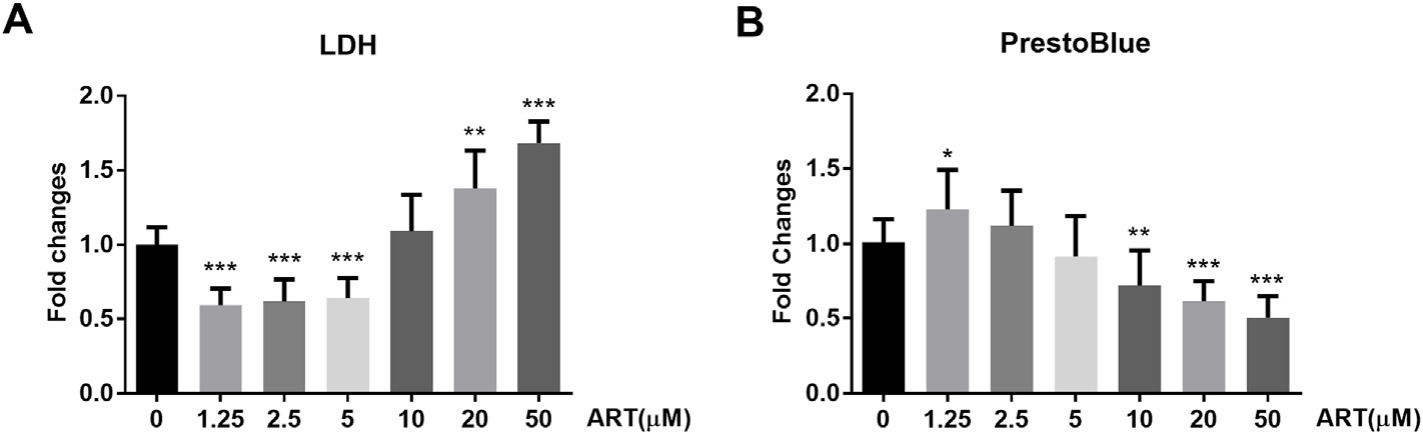
**Effects of ART on the cell toxicity and cell viability in the IMG cells. (A)** Cell toxicity of IMG cells after ART administration for 24 ​h detected by LDH release assay. **(B)** Cell viability of IMG cells after ART administration for 24 ​h detected by PrestoBlue assay. LDH, lactate dehydrogenase; IMG, immortalized microglial cell; ART, artesunate. ∗*P*＜0.05, ∗∗*P*＜0.01, and ∗∗∗*P*＜0.001 versus control.

To find the optimal concentration of ART *in vitro*, IMG cells were treated with ART at various concentrations from 1.25 to 50 ​μM. ART at 1.25 ​μM, 2.5 ​μM, and 5 ​μM reduced LDH release after 24hs treatment, while ART at 20 ​μM and 50 ​μM significantly increased LDH release indicating cell damage at these concentration (∗∗*P* ​< ​0.01, Fig. 1A). In PrestoBlue assay, the viability of the IMG cell was significantly promoted by 1.25 ​μM ART treatment and inhibited ART at concentration over 10 ​μM (∗∗*P* ​< ​0.01, Fig. 1B). Therefore, ART applied below 5 ​μM to IMG cells did not affect the viability with reduction of LDH release.

### 3.2 ART effect on the inflammatory cytokine production by microglial cells

**Fig. 2:**
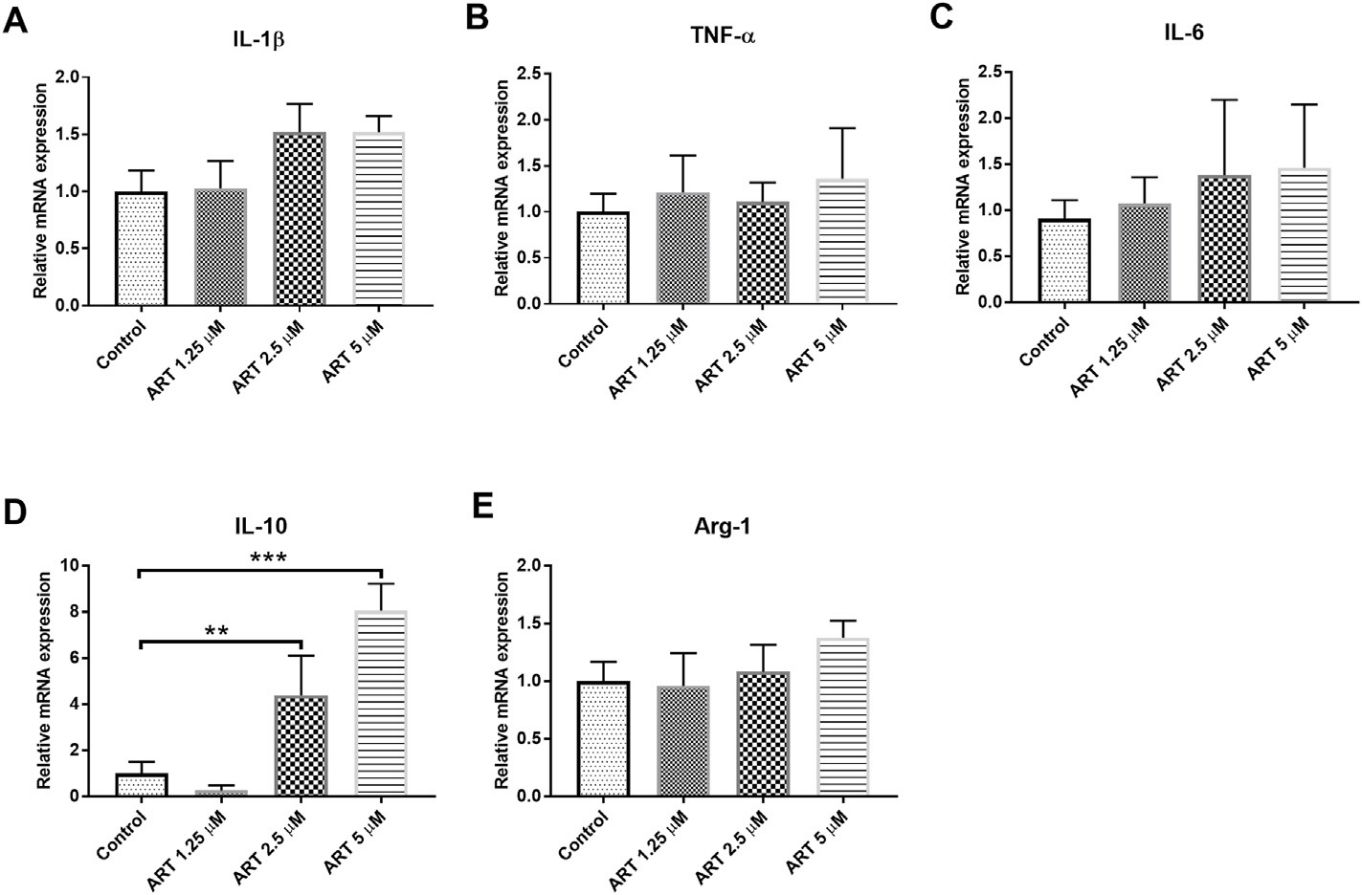
**Effects of ART on the inflammatory cytokine production in the IMG Cells.** IMG cells were administered with ART for 24 ​h. The gene expression levels of IL-1β, TNF-α, IL-6, IL-10, and Arg-1 were determined by RT-PCR (**A-E**). Data are presented as the means ​± ​SD of three independent experiments. n ​= ​4 in each group. ART, artesunate; IL, interleukin; TNF, tumor necrosis factor; Arg, arginase. ∗∗*P*＜0.01 and ∗∗∗*P*＜0.001 versus control.

ART concentrations at 1.25, 2.5, and 5 ​μM that showed no toxic effect, were further used to test the cytokine expression level in the IMG cells when treated for 24 ​h. The gene expressions of pro-inflammatory cytokines (including IL-1β, IL-6 and TNF-α), and anti-inflammatory cytokines (including IL-10 and Arg-1) were detected by RT-PCR. ART at these concentration in the IMG cell culture did not change the expression level of IL-1β, IL-6, TNF-α, and Arg-1 significantly (Fig. 2A–C, and 2E). ART treatment to the IMG cells increased the mRNA expression of IL-10 starting at 2.5 ​μM and reached the statistical significance at 5 ​μM (∗∗*P* ​< ​0.01, Fig. 2D).

### 3.3 Retinal safety assessment of intravitreal ART injection

The *in vitro* study indicated 5 ​μM ART was safe and showed a potential to increase the anti-inflammatory cytokine, IL-10. The concentration of 5 ​μM in cell culture is equivalent to the concentration of 25 ​μM of intravitreal ART injection (1 ​μl) in mice after dilution in vitreous (the vitreous volume of the mouse eye is 5 ​μL).^9^, ^10^, ^11^ To investigate the safety of an intravitreal injection of ART, different doses (25, 50, 100 ​μM, and 1 ​mM) were administrated to the mouse eyes. No ocular inflammation or retinal hemorrhage was observed in the treated eyes. The lens transparency of the mice did not show any obvious changes.

**Fig. 3:**
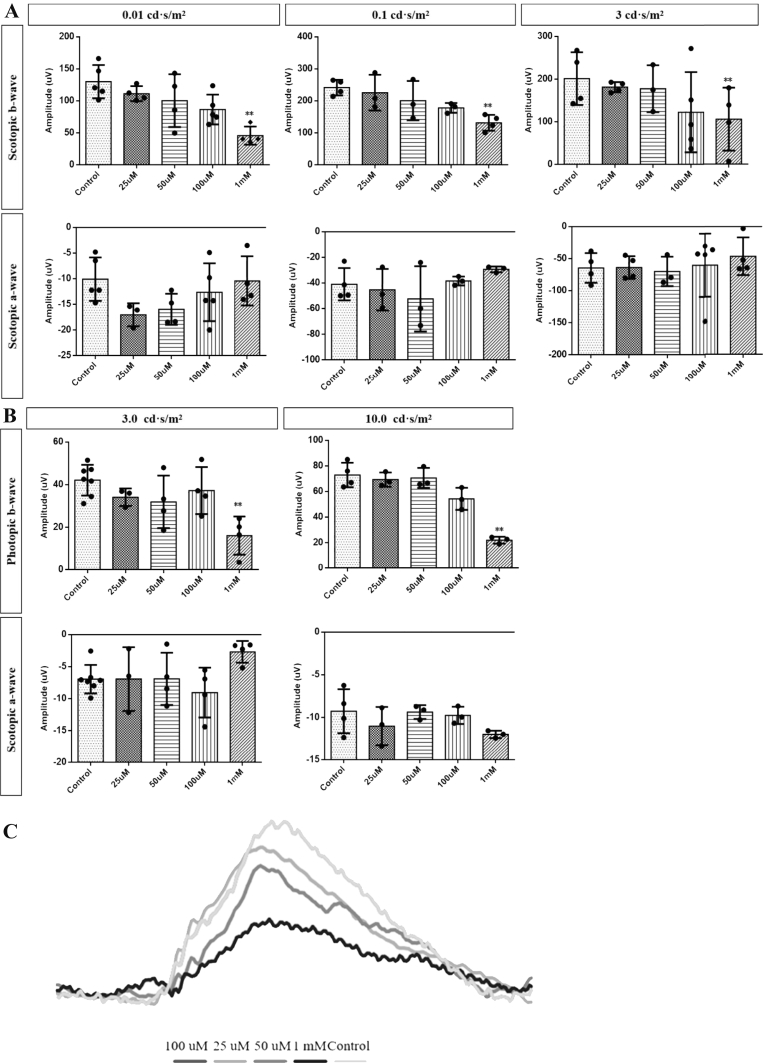
**Effects of ART treatment on the ERG responses of the mouse eyes at 7 days post injection. (A)** The scotopic a- and b-wave amplitudes. **(B)** The photopic a- and b-wave amplitudes. **(C)** Representative scotopic ERG waveforms of each experimental group. ∗∗*P*＜0.01 versus control.

Retinal function detected by flash ERG was carried out at the day 7 after ART treatment. Compared with saline injection eyes, there were no significant changes of retinal function in both scotopic and photopic detection when ART was at 25, 50, and 100 ​μM. High-dose ART (1 ​mM) showed a significant reduction in both scotopic and photopic ERG b-wave (∗∗*P* ​< ​0.01, Fig. 3), indicating the functional deterioration of the inner retina.

**Fig. 4:**
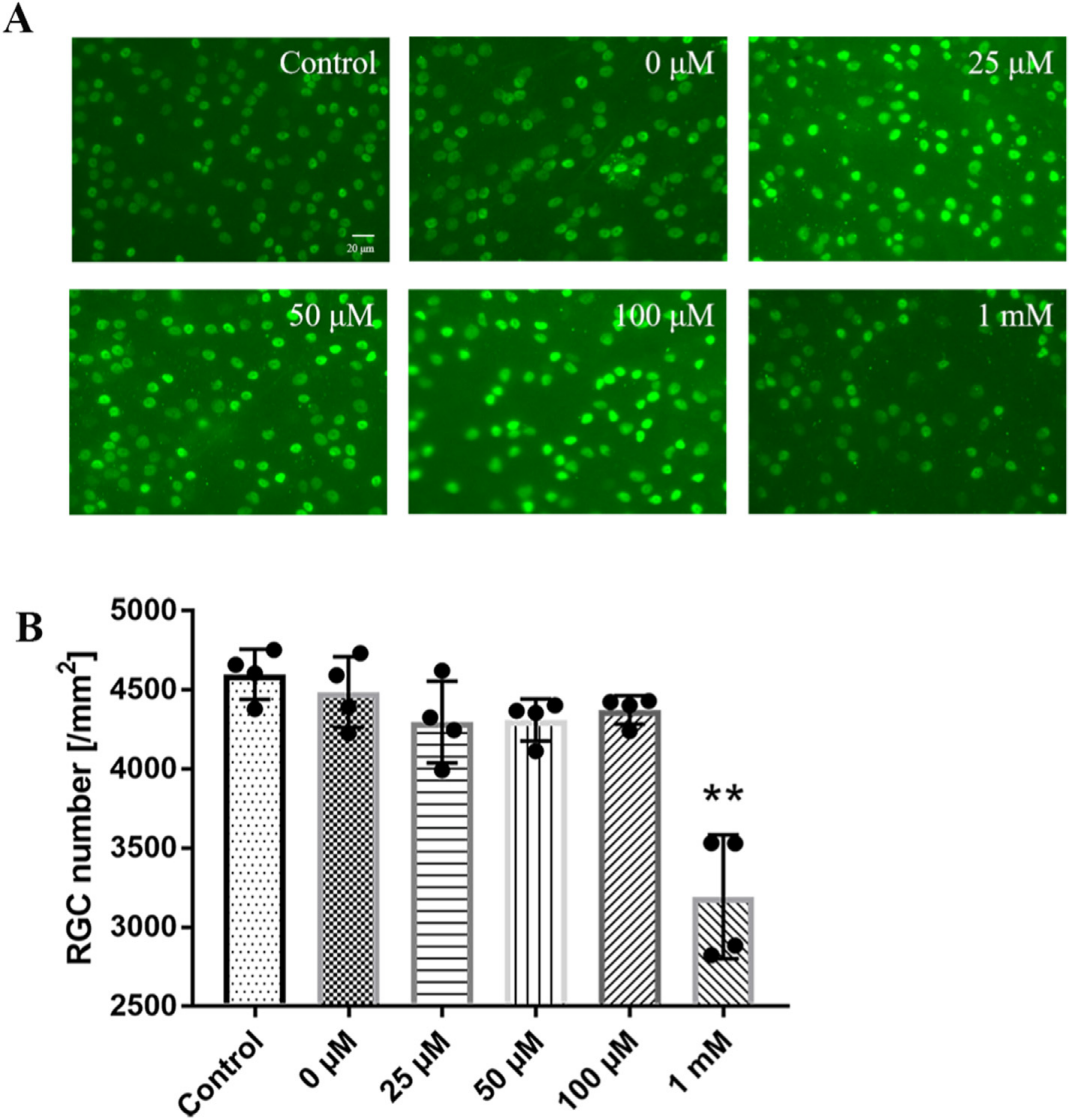
**Effects of ART treatment on the RGC survival in the flat mounted mouse retinas. (A)** Representative images of Brn3a ​+ ​RGC in the normal control eyes and 0, 25, 50, 100 ​μM, and 1 ​mM ART treated eyes. **(B)** RGC densities of the normal control eyes and 0, 25, 50, 100 ​μM, and 1 ​mM ART treated eyes. ∗∗*P*＜0.01 versus control.

The ART effect on the survival of retinal neurons was evaluated by comparing the RGC density on the flat mounted retina. The Brn3a ​+ ​RGC densities (RGCs/mm^2^) of the normal control, saline control, and the ART groups (25, 50, 100 ​μM, and 1 ​mM) were 4599.3 ​± ​158.2, 4486.0 ​± ​222.8, 4296.9 ​± ​259.1, 4310.7 ​± ​132.3, 4374.3 ​± ​89.8 and 3192.8 ​± ​391.8/mm^2^, respectively (Fig. 4). The Brn3a ​+ ​RGC densities of ART groups with low (25 ​μM) and intermediate (50, 100 ​μM) concentrations did not show significant difference versus control (n ​= ​5, *P* ​> ​0.05), while the Brn3a ​+ ​RGC density of the high-dose (1 ​mM) group was significantly reduced (n ​= ​5, ∗∗*P* ​< ​0.01). Consistent with ERG result, intravitreal injection of ART at 25, 50, and 100 ​μM did not cause loss of RGC in the mouse retina, while 1 ​mM ART caused significant RGC loss. Together, the safe range of ART intravitreal injections was 100 ​μM in mouse.

### 3.4 ART effect on the inflammatory cytokine production in the mouse retina

**Fig. 5:**
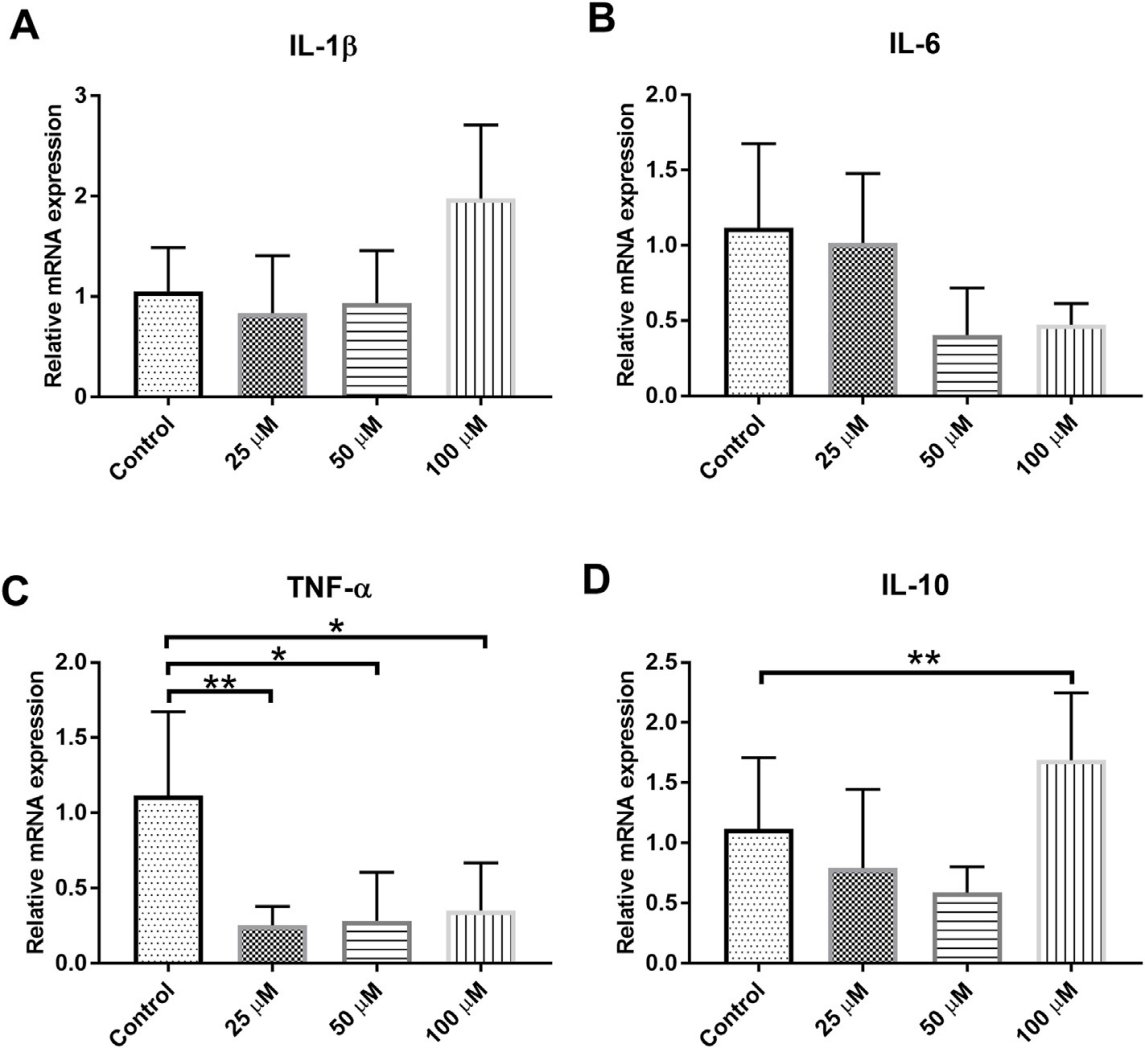
**Effects of ART on the inflammatory cytokine production in the mouse retina.** The gene expression levels of IL-1β, IL-6, TNF-α, and IL-10 in the normal control eyes and 25, 50, and 100 ​μM ART treated eyes were determined by RT-PCR (**A-D**). Data are presented as the means ​± ​SD of three independent experiments. n ​= ​4 in each group. ART, artesunate; IL, interleukin; TNF, tumor necrosis factor. ∗*P*＜0.05, ∗∗*P*＜0.01 versus control.

To verify the observations of cytokine changed induced by ART on the IMG cells, the mRNA expression of IL-1β, IL-6, TNF-α, and IL-10 in the retina after 7 days of ART intravitreal injection were detected. Compared to the saline control, ART at 25, 50 and 100 ​μM significantly reduced TNF-α expression (∗*P* ​< ​0.05, ∗∗*P* ​< ​0.01, Fig. 5C); ART at 50 and 100 ​μM also reduced IL-6 expression (Fig. 5B). ART at 100 ​μM significantly increased IL-10 expression (∗∗*P* ​< ​0.01, Fig. 5D) and elevated the expression of IL-1β.

## 4 Discussion

In the current study, the safe doses of ART were investigated both *in vitro* and *in vivo*. ART at 5 ​μM was safe for IMG cells *in vitro* and even provided a minor protective effect at 24 ​h post treatment. The viability of IMG cells was reduced by 10 ​μM ART, and cell toxicity was induced by 20 ​μM ART. When injected into the mouse vitreous, the retinal function (b-wave in flash ERG test) and RGC survival were not significantly affected at ART concentration of 25, 50, and 100 ​μM (1 ​μl injection). Toxic effect of intravitreal ART was at 1 ​mM. Moreover, we found that 5 ​μM ART significantly promoted the gene expression of IL-10 in IMG cells while 100 ​μM ART intravitreal injection downregulated TNF-α and upregulated IL-10 in the mouse retina, indicating a potential anti-inflammation of ART in ophthalmic application.

In 2022, Qin et al. reported that reduced the cell viability detected by MTT method, ART at 1 ​μM in BV2 microglial and N-2a neuron in cell culture.^6^ Our study tested if ART would affect microglial survival in another cell line, IMG. The culture condition of the BV2 microglia during ART treatment was in high FBS. In current IMG culture during ART treatment, low glucose low FBS condition was adopted to restrict the environmental effect on cell survival with the emphasis on ART. In Qin's study, MTT assay was adopted to detect viability of both microglia and neuronal cells, we used LDH to detect cell death and Prestoblue assay for cell viability detection. Both cell culture medium and cells from the same well were analyzed at the same time, the result reflexes better for the entire profile of the same treatment. Treated with ART, the IMG cell viability was increased at 1.25 ​μM and started to decrease at the concentration of 10 ​μM. This is consistent with the result from LDH detection which showed increased cell death at 10 ​μM. ART effect on the microglia might be complicated when microglia-neuron interaction is taken into consideration in the *in vivo* situation. To evaluate whether ART imposed any acute toxic effect on the retinal neurons and adverse retinal glial activation, the evaluation was set at 7 days post intravitreal injection. This is the time point when acute insult to the retina causes significant decreased retinal function and loss of RGC, such as under retinal ischemia reperfusion and optic nerve axotomy condition. There is no significant restriction to small molecule drug diffusion in the vitreous humor as compared to water.^11^ Thus, when the 1 ​μL ART solution was injected into the vitreous cavity, it would rapidly diffuse into the vitreous humor and reach the surface of RGC. The vitreous volume of the mouse eye is about 5 ​μL, 1 ​μL ART injection was diluted by 5 times.^10^ To investigate the safety of an intravitreal injection of ART, different doses (25, 50, 100 ​μM, and 1 ​mM) were administrated to the mouse eyes. This testing range covered the dose used in the human study using 80 ​μg ART for intravitreal injection.^5^ The volume of human vitreous cavity is about 4.5 ​ml, and the final concentration of the ART equals to 46.2 ​μM. Our study using both retinal functional test and RGC cell counting confirmed that the maximum ART concentration can be used is 100 ​μM intravitreally. This further increased the safety range of our previous study in which intravitreal injection of 20 ​μg/ml ART (about 50 ​μM) was used in SD rats.^9^

The cytokine expression regulated by ART treatment is in consistence with previous reports about the neuroprotective effects of low dose artemisinin in a mouse model of multiple sclerosis.^12^^,^^13^ Low-dose artemisinin could downregulate Th1 responses, decrease the production of pro-inflammatory cytokines, and induce the production of Th2-type cytokines more significantly than the high-dose artemisinin^12^. In retinal vascular disorders, angiogenesis and inflammation have been shown to be involved in the pathogenesis. The homeostatic state of microglia is essential to regulate the innate immune system throughout retinal disorders.^14^ To examine if ART treatment has effects on the inflammatory responses of IMG cells, the mRNA expression levels of related cytokines were evaluated. We found that the mRNA expressions of pro-inflammatory factors including IL-1β, IL-6, and TNF-α remained unchanged after ART treatment while IL-10 expression was remarkably elevated by ART administration at the concentration of 2.5 ​μM and 5 ​μM in IMG cells. Additionally, the mRNA expression of TNF-α was downregulated while IL-10 was upregulated in the mouse retina treated with 100 ​μM ART. Previous studies have illustrated the potential anti-inflammatory property of ART under a variety of circumstances, such as systemic lupus erythematous, allergic asthma, arthritis, uveitis, and ulcerative colitis.^15^, ^16^, ^17^, ^18^, ^19^, ^20^ However, the underlying mechanisms of its immunomodulatory effects remain unclarified. It is well-recognized that microglia play an important role in maintaining immune homeostasis, which can be polarized into M1 phenotype to release pro-inflammatory factors including TNF-α, IL-1β, IL-6, and M2 phenotype to up-regulate anti-inflammatory factors such as IL-10, Arg-1, if disturbed.^21^^,^^22^ ART (0.5–4μM) has been shown to significantly inhibit TNF-α and IL-6 production, reduce NF-κB-driven luciferase expression and inhibit IκB phosphorylation as well as degradation in activated BV2 microglia.^23^ One recent study also demonstrated that ART was able to reduce traumatic brain injuries (TBI) - induced lesion through the inhibition of microglia and astrocytes activation.^24^ Anti-inflammatory action was verified through the inhibition of NF-κB, whereas the release of pro-inflammatory cytokines such as TNF-α and IL-1β was shown.^24^ Therefore, our RT-PCR findings provide evidence for the anti-inflammation of ART, rendering a potential therapeutic approach for ophthalmic disorders.

## 5 Conclusions

Our study showed that ART is safe when administered at the concentration of no more than 100 ​μM (1 microliter in volume) in the normal mouse eye. Intravitreal injection of 100 ​μM ART could downregulate TNF-α while upregulate IL-10 in the mouse retina without causing retinal functional deterioration and RGC loss. ART might be used as anti-inflammatory agent for retinal disorders. Its anti-inflammatory potential remains an interesting topic that warrants further study in ophthalmic practices.

## Study approval

The authors confirm that any aspect of the work covered in this manuscript that involved human patients or animals was conducted with the ethical approval of all relevant bodies and the ​study was performed in accordance with the Declaration of Helsinki, and the protocol was approved by the Ethics Committee on the Use of Live Animals in Teaching and Research, LKS Faculty of Medicine The University of Hong Kong (approval number: CULATR 5364-20).

## Author contributions

Lu BW contributed to Design, Data curation, Formal analysis, Investigation, Methodology, and Drafting the Manuscript; Liang YX, investigation, methodology (iv injection); Liu JF, investigation, methodology (mRNA detection) and manuscript editing/Revising; Sun ZQ, investigation, methodology. So KF, investigation, manuscript editing; Chiu K: Conceptualization and design, Resources, Data curation, Investigation, Supervision, Project administration and Editing/Revising the manuscript. All authors have read, commented, and approved the final content of the manuscript.

## Funding

This work was supported by Midstream Research Programme for Universities, Hong Kong to Kin Chiu (Project No: MRP-092–17X).

## Declaration of competing interest

The authors declare that they have no known competing financial interests or personal relationships that could have appeared to influence the work reported in this paper.

## Abbreviations

ART: Artesunate
IMG: Immortalized Microglial
RGC: Retinal Ganglion Cell
NV: Neovascularization
ME: macular edema
DMEM: Dulbecco's modified Eagle's medium
FBS: Fetal Bovine Serum
LDH: Lactate Dehydrogenase
